# The Successful Treatment of a Case of Linear Psoriasis with Ixekizumab

**DOI:** 10.1155/2017/3280215

**Published:** 2017-11-02

**Authors:** Sara Ghoneim, Alvaro J. Ramos-Rodriguez, Fernando Vazquez de Lara, Lauren Bonomo

**Affiliations:** ^1^Saba University School of Medicine, The Bottom, Netherlands; ^2^Icahn School of Medicine at Mount Sinai West, New York, NY, USA; ^3^Icahn School of Medicine at Mount Sinai, New York, NY, USA

## Abstract

Linear psoriasis is an unusual clinical variation of psoriasis that manifests segmentally along the lines of Blaschko. A major differential diagnosis is inflammatory linear verrucous epidermal nevus (ILVEN). The treatment of linear psoriasis is often challenging, with inadequate response to biological agents reported in the literature. We report a case of a 25-year-old African-American female who presented with asymptomatic hyperkeratotic papules along the lines of Blaschko and was subsequently diagnosed with linear psoriasis. After failing conventional treatment regimens, the patient received a trial of ixekizumab with complete resolution of cutaneous lesions reported after 4 months and only 8 doses of the anti-IL-17 biologic agent.

## 1. Introduction

Linear psoriasis is a rare clinical variation of psoriasis that manifests segmentally along the lines of Blaschko. The pathogenesis remains unclear, though some have proposed it could be explained by the well-established concept of genetic mosaicism [1]. Happle (1991) suggested that the loss of heterozygosity in somatic cells during early embryogenesis results in somatic recombination with daughter cells. Subsequently, these daughter cells go on to become clonal stem cells proliferating in a linear pattern during the embryonic development of the skin. A major differential diagnosis for linear psoriasis is inflammatory linear verrucous epidermal nevus (ILVEN), which also presents along the lines of Blaschko with similar morphology [2]. Psoriasis presenting in this manner is often mistaken for ILVEN and undertreated. The treatment of linear psoriasis can be challenging, with reports of inadequate clinical response to various biologic agents approved for the treatment of plaque psoriasis [3]. To our knowledge, we report the first case of this atypical psoriasis morphology successfully treated with the biologic agent ixekizumab.

## 2. Case Report

A 25-year-old African-American female presented to our clinic with asymptomatic lesions linearly arranged over her left upper extremity. The initial lesion first appeared fifteen years ago and new lesions gradually appeared over time. She denied joint pain and/or a history of infections prior to lesion development. Her past medical history was significant only for posttraumatic distress disorder and depression. There was no personal or family history of psoriasis or other dermatologic disease. Prior to presentation in our clinic, she had a skin biopsy of the right forearm which showed chronic spongiotic dermatitis with parakeratotic foci and superficial perivascular mononuclear infiltrates. No deep dermal or periadnexal infiltrates were seen and periodic acid-Schiff staining was negative for fungal organisms. Based on the results, both lichen striatus and linear psoriasis were considered as potential diagnosis, and she was started on high-potency topical steroids. A month later, the patient was referred to our clinic when she failed to respond to treatment.

**Figure 1:**
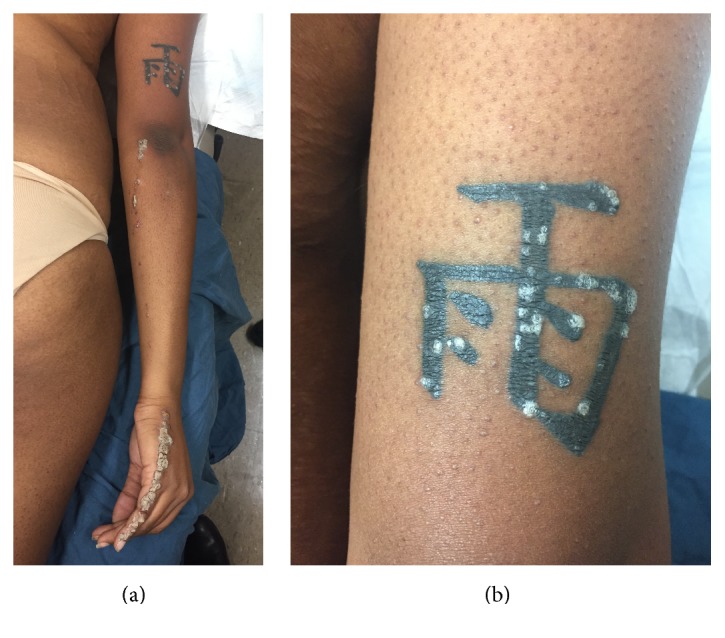
(a) Linearly arranged hyperkeratotic and scaly gray papules on the right fifth finger and dorsum of the hand extending to the right elbow. (b) Multiple hyperkeratotic papules present within a tattoo on the posterior right arm.

**Figure 2:**
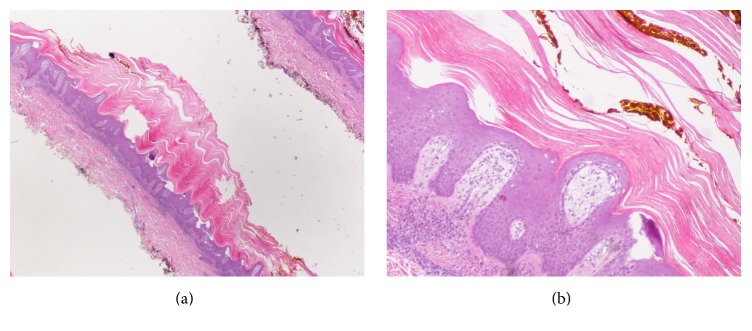
(a) Histopathological slides. At 2x magnification there is parakeratosis and epidermal acanthosis. (b) Histopathological slides. At 10x magnification a regularly acanthotic epidermis with hyperkeratosis alternating with parakeratosis. Rete ridges show psoriasiform hyperplasia.

**Figure 3:**
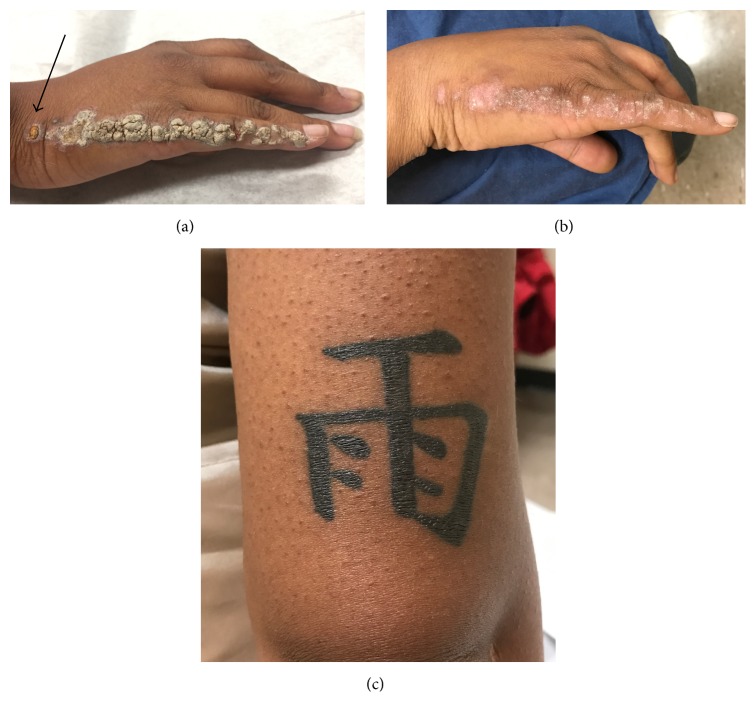
(a) Close-up of lesions present on right fifth finger and dorsum of hand. At two-week follow-up, new papules appear in the area that is previously electrodessicated (black arrow). (b) Remarkable clearing of the lesions and postinflammatory hypopigmentary changes can be seen after 8 doses of ixekizumab. (c) Psoriatic lesions are no longer present on the tattoo after treatment with 8 doses of ixekizumab.

Physical examination revealed hyperkeratotic and scaly gray papules coalescing into a linear plaque of the right dorsal fifth finger extending medially to the right elbow (Figure 1(a)). Of note, scaly papules were also present on a tattoo above the right elbow (Figure 1(b)). There was no nail or palmoplantar involvement. The differential diagnosis included linear psoriasis and ILVEN. The isomorphic reaction seen within the patient's tattoo (Koebner phenomenon) favored a diagnosis of psoriasis. A skin biopsy and electrodessication of one papule on her right dorsal fifth finger were performed. Histological examination of the specimen revealed parakeratosis with uniformly acanthotic epidermis (Figure 2). At a follow-up visit 2 weeks later, the patient developed new papules with similar morphology in the area that was previously electrodessicated (Figure 3(a)). This new episode of Koebnerization and histological findings further supported our diagnosis of linear psoriasis and the decision was made to initiate treatment with ixekizumab, a monoclonal antibody targeting interleukin- (IL-) 17A. Four months later and after 8 doses of ixekizumab, we observed almost-complete resolution of the cutaneous lesions (Figures 3(b) and 3(c)).

## 3. Case Discussion

Linear psoriasis is a rare clinical presentation of psoriasis characterized by the linear distribution of psoriatic lesions along the lines of Blaschko. The main differential diagnosis is ILVEN. Gross morphological distinction between these two entities is difficult. Furthermore, the two entities share similar histological findings and the coexistence of ILVEN and psoriasis has also been reported [3, 4]. Histological examination of ILVEN shows areas of hypergranulosis and orthokeratosis alternating with areas of hypogranulosis and parakeratosis [5]. Under these circumstances, immunohistochemical studies are helpful in distinguishing these two cutaneous disorders. For example, involucrin is a marker whose expression is absent in the parakeratotic areas of ILVEN but present in psoriasis [6]. Moreover, the number of Ki-67 positive nuclei is higher in psoriasis compared to ILVEN, while the number of keratin-10 positive cells is higher in ILVEN [7]. In practice, these tests are rarely ordered. It is often more practical to simply initiate therapy for psoriasis if the diagnosis is suspected.

We are now able to conclude that linear psoriasis was the correct diagnosis in this case based on several observations. First, our patient's lesions were nonpruritic, and ILVEN tends to be more pruritic than psoriatic lesions [8]. Additionally, Koebner phenomenon affects 25–30% of patients with psoriasis [9], and our patient developed lesions on her tattoo and on the electrodessicated site of her right dorsal hand. To our knowledge, Koebner phenomenon has not been described in any reported case of ILVEN. Finally, ILVEN responds minimally to antipsoriatic agents, and our patient had a remarkable response to only 8 doses of the biologic agent ixekizumab, a drug approved by the US Food and Drug Administration in 2016 for the treatment for moderate to severe plaque psoriasis.

It is known from a small number of reports that segmental manifestations of psoriasis respond less favorably to systemic therapies such as methotrexate, acitretin, and, more recently, biologics [3, 10–13]. The chronicity and resistance of linear psoriasis to available antipsoriatic agents were suggested to be in part due to the loss of heterozygosity in cells where the lesions occur [12]. In our literature review, patients treated with either anti-IL-23 or tumor necrosis-alpha inhibitor agents had significant improvements on all of the types of psoriasis except in linear psoriasis (Table 1). Multiple studies have also demonstrated that even though psoriatic lesions might look similar, they differ substantially in the activation status of inflammatory and cytokine pathways and such networks might contribute to the different treatment responses observed with biologic agents [14]. Furthermore, by analyzing psoriasis transcriptosome in nontreated biopsied lesions, one study was able to differentiate between etanercept responders and nonresponders [15]. This heterogeneity in response further underscores the potential role of gene-expression profiling as potential predictors of response to biologics. There are no current formal guidelines for the treatment of linear psoriasis, and larger studies are needed to determine optimal therapy for this rare variant. In our case, the patient responded favorably to ixekizumab, which opens the possibility of using new biologic agents and individualized therapy in patients with recalcitrant linear psoriasis.

## Figures and Tables

**Table 1:** Summary of reported cases of linear psoriasis treated with a biological agent.

Authors (year)	Gender	Age	Distribution of linear psoriasis (LP)	Other features	Biological agent used and outcome
Colombo et al. (2011) [3]	Male	67 years	Middle of ventral trunk and left side of arm, hand, thigh, knee, and tibia	Psoriatic arthritis and diffuse plaque psoriasis. Failed to respond to acitretin, cyclosporine, and methotrexate	Plaque psoriasis responded to *etanercept* but not LP

Rott et al. (2007) [10]	Male	11 years	Left side of the body	Psoriatic arthritis, nail changes. Failed methotrexate, cyclosporine and etanercept	Psoriatic arthritis responded to *infliximab* but not LP

Sfia et al. (2009) [11]	Male	29 years	Left arm and left leg	Additional psoriatic plaques on the body	Psoriatic plaques responded to *infliximab* but not LP

Arnold et al. (2010) [12]	Male	50 years	Left flank	Diffuse plaque psoriasis. Failed to respond to topical steroids, PUVA, UVB, cyclosporine, and etanercept	Plaque psoriasis responded to *adalimumab* but not LP

Weng and Tsai (2017) [13]	Male	27 years	Right upper arm, shoulder, and back	In addition to plaque psoriasis. Failed to respond to methotrexate, acitretin, topical vitamin D3 analogs and steroids	Plaque psoriasis responded to *ustekinumab* but not LP

Ghoneim et al. (2017)	Female	25 years	Dorsum of right hand, forearm and arm, and suprapubic region, left thigh and occiput	Failed topical high-potency steroids	Linear psoriasis responded favorably to 8 doses of *ixekizumab*
